# Supplementary material for: Biomechanical Properties of Strictures in Crohn’s Disease: Can Dynamic Contrast-Enhanced Ultrasonography and Magnetic Resonance Enterography Predict Stiffness?
Source: Diagnostics (Basel). 2022 Jun 2;12(6):1370. doi: 10.3390/diagnostics12061370 (PMC9221822; doi:10.3390/diagnostics12061370)
Supplement: Supplementary file 1 [file diagnostics-12-01370-s001.zip › diagnostics-1715816-supplementary.pdf]

Figure S1. Regions of Interests

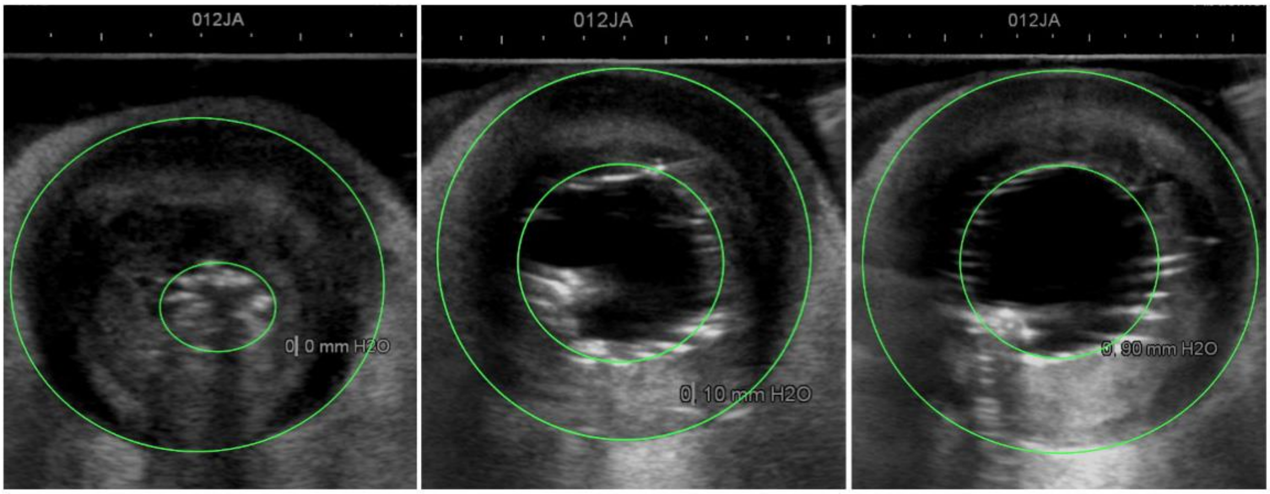

Regions of interests drawn in OsiriX. Patient 012 at location “0” at 0, 10 and 90 cmH2O. On US falsely labeled mm instead of cm

Table S1

Demographics	
Parameter	No. of Patients
Included patients	18
Females	10 (56 %)
Age, years	43 [19-66]
Body mass index (kg/m^2)	25.0 [20.2-32.0]
Disease duration	8.8 [0-25.3]
< 2 years	5 (28 %)
2-10 years	6 (33 %)
>10 years	7 (39 %)
Location of disease	
L1, Terminal Ileum	12 (67 %)
L2, Colon	0 (0 %)
L3, Ileocolon	5 (28 %)
L4, Upper disease	1 (6 %)
Crohn Disease Activity Index	210 ± 94
Harvey Bradshaw Index	8.0 ± 4.6
Fecal Calprotectin (µg/g)*	251 [49-1422]
C-Reactive Protein (mg/l)*	4.3 [0.6-29.4]
Hemoglobin (mmol/l)	8.9 ± 0.7
Albumin (g/l)	36.3 ± 3.8
Vitamin D (nmol/l)	69.5 ± 20.8
Hematocrit	0.42 ± 0.04
Time between examinations, days*	O [0-4]
Time between US and Surgery, days*	7.5 [1-26]
Symptoms within last flair, n (%), days*	
Pain	16 (89 %)
Nausea	13 (72 %)
Vomit	6 (33 %)
Diarrhea	11 (61 %)
Bloody stools	5 (28 %)
Bloating	12 (67 %)
Weight loss	8 (44 %)
Fatigue	3 (17 %)
IBD Treatment, n (%)	
None	5 (28 %)
Steroids	5 (28 %)
Immunosuppressors	3 (17 %)
Biological therapy	4 (22 %)
Combo treatment	1 (6 %)

Note – Numbers in parenthesis are percentages. Numbers in brackets are ranges. Unless indicated otherwise, data are means ± standard deviations. * Median values and ranges. US = Ultrasonography

Table S2

Correlation between Young's modulus and imaging

Parameter	Spearman's rho	P value
CEUS		
Peak Enhancement	0.00	P = .99
Area Under Curve	-0.04	P = .89
Wash-in Rate	0.08	P = .76
Wash-in Perfusion index	0.00	P = .99
Rise Time	-0.22	P = .41
Fall Time	-0.09	P = .74
DCE-MRE		
Peak Enhancement	0.24	P = .35
Area Under Curve 70 s	-0.04	P = .89
Initial Slope	0.63	P = .007
Initial Slope Max	0.30	P = .25
Wash-in Perfusion Index	0.03	P = .90
Rise Time	-0.33	P = .19
Wash-out Slope 60 s	0.10	P = .71
Wash-out Slope 120 s	-0.17	P = .52

Note – § P value for One-way analysis of variance.
CEUS = Contrast enhanced ultrasonography, DCE-MRE = Dynamic Contrast Enhanced MR Enterography.

Figure S2. Correlation with MR enterography global score

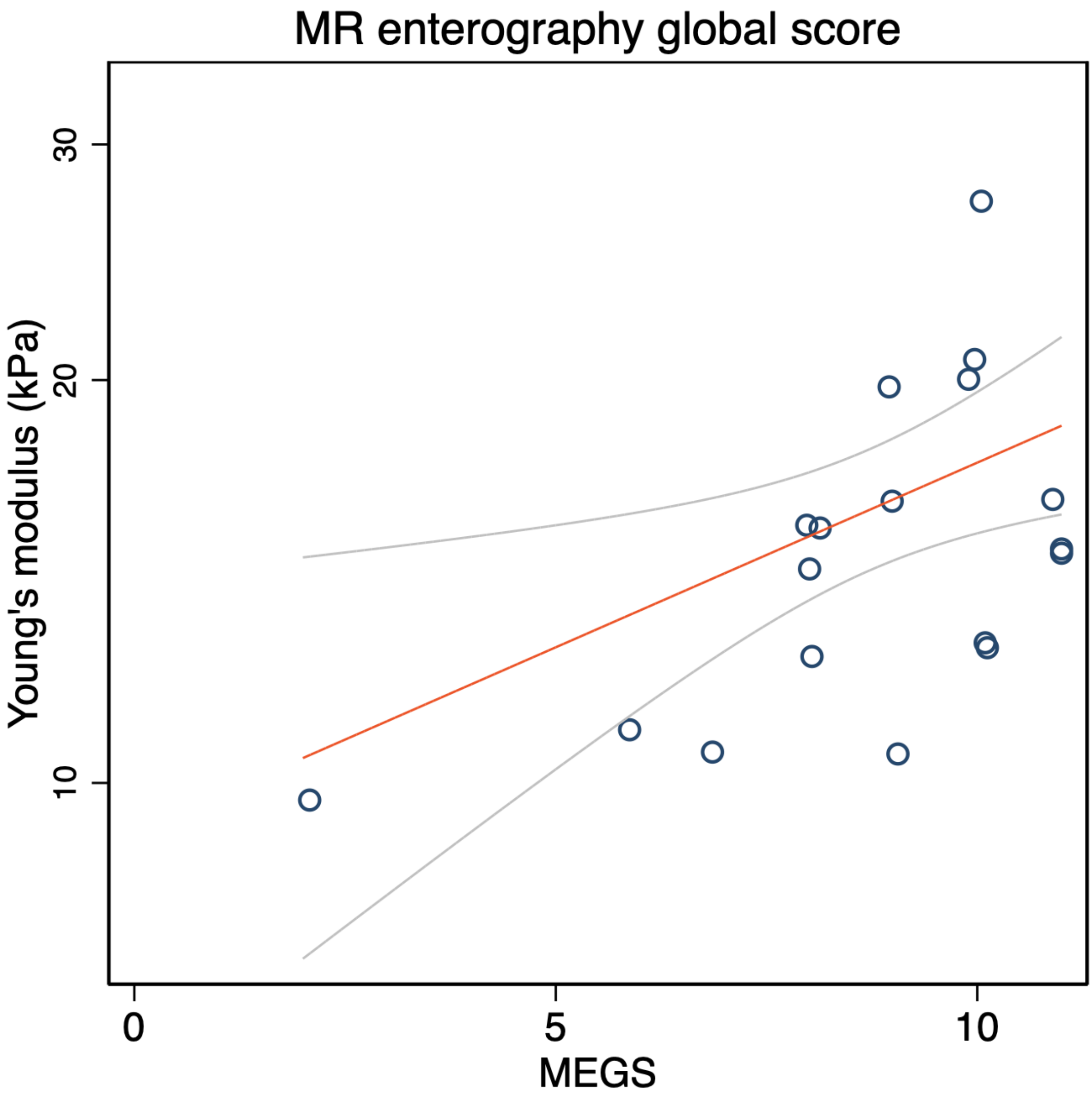

Association between stiffness of the stricture and MR enterography global score (MEGS) (Spearman's ρ=0.55, P=0.018).
